# TDP43 pathology in chronic traumatic encephalopathy retinas

**DOI:** 10.1186/s40478-023-01650-6

**Published:** 2023-09-22

**Authors:** Ragini Phansalkar, Vanessa S. Goodwill, Jeffrey J. Nirschl, Chiara De Lillo, Jihee Choi, Elizabeth Spurlock, David G. Coughlin, Donald Pizzo, Christina J. Sigurdson, Annie Hiniker, Victor E. Alvarez, Ann C. Mckee, Jonathan H. Lin

**Affiliations:** 1 School of Medicine, Stanford University, Stanford, CA USA; 2 Department of Pathology, University of California, San Diego, La Jolla, CA USA; 3 Departments of Ophthalmology and Pathology, Stanford University, Stanford, CA USA; 4 Vita-Salute San Raffaele University, Milan, Italy; 5 The University of Texas Southwestern Medical Center, Dallas, TX USA; 6 Boston University Alzheimer’s Disease and CTE Center, Boston University School of Medicine, Boston, MA USA; 7 Department of Neurosciences, University of California, San Diego, La Jolla, CA USA; 8 VA Boston Healthcare System, Boston, MA USA; 9 VA Palo Alto Healthcare System, Palo Alto, CA USA

**Keywords:** Chronic traumatic encephalopathy (CTE), Retina, Inner nuclear layer, Tau, TDP43, Eye pathology

## Abstract

Chronic traumatic encephalopathy (CTE) is a neurodegenerative disease associated with repetitive head trauma. Brain pathology in CTE is characterized by neuronal loss, gliosis, and a distinctive pattern of neuronal accumulation of hyper-phosphorylated tau (p-tau) and phospho-TDP43 (p-TDP43). Visual anomalies have been reported by patients with CTE, but the ocular pathology underlying these symptoms is unknown. We evaluated retinal pathology in post-mortem eyes collected from 8 contact sport athletes with brain autopsy-confirmed stage IV CTE and compared their findings to retinas from 8 control patients without CTE and with no known history of head injury. Pupil-optic nerve cross sections were prepared and stained with hematoxylin and eosin (H&E), p-tau, p-TDP43, and total TDP43 by immunohistochemistry. No significant retinal degeneration was observed in CTE eyes compared to control eyes by H&E. Strong cytoplasmic p-TDP43 and total TDP43 staining was found in 6/8 CTE eyes in a subset of inner nuclear layer interneurons (INL) of the retina, while only 1/8 control eyes showed similar p-TDP43 pathology. The morphology and location of these inner nuclear layer interneurons were most compatible with retinal horizontal cells, although other retinal cell types present in INL could not be ruled out. No p-tau pathology was observed in CTE or control retinas. These findings identify novel retinal TDP43 pathology in CTE retinas and support further investigation into the role of p-TDP43 in producing visual deficits in patients with CTE.

## Introduction

Chronic traumatic encephalopathy is a progressive neurodegenerative disorder associated with repetitive head trauma that has been found in the brains of athletes who compete in contact sports, such as football, wrestling, ice hockey, and soccer [22, 23, 25, 29]. CTE has also been identified in individuals exposed to military-related traumatic brain injury (TBI), interpersonal violence, poorly controlled epilepsy, and other sources of repetitive head impacts (RHI) [7, 11, 18, 27].

Common presenting symptoms of CTE include behavioral disturbances, depression, and short-term memory loss, with dementia common in severe cases [28, 30]. The disease progresses to cause more severe cognitive impairment, behavioral changes, suicidality, and in some cases motor deficits. Visuospatial anomalies have been reported as a common symptom in CTE [28], but the specific visual abnormalities have not been well-described. Additionally, a variety of visual changes have also been reported post-TBI, including photophobia, double-vision, and abnormalities in saccades, convergence, and accommodation [33, 36]. However, the anatomical basis and ocular pathology that cause visual symptoms in TBI and CTE are unknown.

CTE is defined by its distinctive p-tau pathology consisting of a perivascular accumulation of hyper-phosphorylated tau (p-tau) in neurons as neurofibrillary tangles and neurites at the depths of the cortical sulci [1, 4, 16, 24, 25, 28]. As disease severity progresses, p-tau accumulation is observed throughout the cortex, medial temporal lobes, and diencephalon [28]. The severity of CTE has been classified into four stages with stage I and II considered mild and stages III and IV considered severe [28]. Stage IV is the most severe CTE stage and typically shows widespread p-tau pathology throughout the brain, including the spinal cord, basis pontis, and dentate nucleus of the cerebellum, in addition to marked neuronal loss and gliosis in the cortex and medial temporal lobe structures. In addition to a distinctive p-tau pathology, many cases of CTE also show progressive accumulation of phosphorylated Transactive Response DNA-binding protein 43 (p-TDP43) in the white matter and cortex [28, 32]. Immunopositivity for p-TDP43 in CTE takes the form of neurites, as well as intraneuronal and intraglial inclusions [8, 28]. The first NINDS-NIBIBS consensus panel found TDP43 pathology to be distinctive in CTE and considered it a supportive feature of CTE diagnosis [24].

No previous studies have examined ocular pathology or p-tau and TDP43 deposition in the eyes of individuals with CTE. In contrast, abnormal protein accumulations have been identified in the retina in other neurodegenerative disorders, such as prion protein in Creutzfeldt-Jakob disease [12, 13, 35], and amyloid beta and p-tau deposits in some cases of Alzheimer’s disease [9, 17] (but not in other retrospective studies [14, 37]). Here, we evaluate the histopathology of post-mortem eyes collected from 8 patients with autopsy-confirmed stage IV CTE, as well as 8 autopsy controls, for evidence of neurodegeneration, p-tau, and p-TDP43 accumulation.

## Materials and methods

### Ethical approval and subject selection

This study was approved by independent institutional review boards at Boston University, UC San Diego (IRB #131208), and Stanford University (IRB #54760). In each case, written informed consent was obtained from subjects and the next of kin with authorization for a diagnostic autopsy with donation of brain tissue and globes. The 8 CTE enucleation specimens came from patients with clinical histories of repetitive sports-related head impacts and stage IV CTE brain neuropathology upon autopsy (Table 1). The 8 control enucleation specimens came from individuals with no known history of TBI or exposure to RHI, and no evidence of CTE neuropathology at autopsy (Table 2). In the CTE cases, CTE stage was determined to be stage IV using the McKee criteria [1], and “High” CTE using NINDS consensus criteria [4, 24].

**Table 1:** Clinical features of pathology-confirmed CTE cases

No.	Age	Sex	Primary pathologic diagnosis	Cause of death	Athletic history	Position played	No. of concussions	p-TDP43 presence	p-TDP43 positive regions
1	86	M	CTE Stage IV	Not available	Semi-professional football	Not available	Not available	Unknown	Unknown
2	80	M	CTE Stage IV	Respiratory failure	NFL	Running back	1 (0 LOC)	Yes	Spinal cord, Amygdala, Hippocampus, Entorhinal cortex/Inferior temporal cortex, Neocortex
3	93	M	CTE Stage IV	Dementia related	Amateur boxing	Not applicable	Unknown	Yes	Spinal cord, Amygdala, Hippocampus, neocortex
4	82	M	CTE Stage IV	Respiratory failure	NFL	Defensive back, safety, special teams	~ 100 (unknown LOC)	Yes	Amygdala, hHppocampus, Entorhinal cortex/Inferior temporal cortex
5	77	M	CTE Stage IV	Cardiac condition	Australian Rugby	Flanker, break-away	“1000 s” (0 LOC)	Yes	Spinal cord, Amygdala, Hippocampus
6	70	M	CTE Stage IV	Dementia related	“Professional football”	Defensive back, safety	10 (1 LOC)	Yes	Spinal cord, Amygdala, neocortex
7	62	M	CTE Stage IV	Cardiac condition	NFL	Defensive back, safety	25 (1 LOC)	Yes	Amygdala, Hippocampus
8	70	M	CTE Stage IV	Cardiac condition	NFL, recreational rugby	Offensive lineman	10 (1 LOC)	Yes	Amygdala, Hippocampus, Entorhinal cortex/Inferior temporal cortex

CTE, chronic traumatic encephalopathy; NFL, National Football League; No., number; LOC, loss of consciousness

**Table 2:** Clinical features of control cases

No	Age	Sex	Neuropathologic diagnosis	Cause of death	p-TDP43 presence in brain	Eye path findings
1	81	M	Basilar artery atherosclerosis	Acute myocardial infarction	Unknown	Scleral buckle; intraocular lens
2	61	M	Metabolic encephalopathy	Septic shock	Unknown	Numerous drusen
3	68	M	NSA	Acute bronchopneumonia	Unknown	NSA
4	72	F	Alzheimer’s disease changes (mild to moderate)	Metastatic adenocarcinoma	Unknown	Scattered drusen
5	70	M	Alzheimer’s disease changes (mild), metabolic encephalopathy	Bronchopneumonia	Negative in hippocampus	Scattered drusen
6	76	M	Terminal acute ischemic changes, amyloid angiopathy, intracortical microinfarct	Lymphocytic myocarditis	Negative in hippocampus	Scattered drusen
7	68	F	NSA	Ischemic cardiomyopathy	Negative in hippocampus	NSA
8	61	M	Cerebrovascular disease	Cardiac condition	Unknown	NSA

No., number; NSA, no significant abnormalities

### Histology and immunohistochemistry

All autopsy globes were fixed in 10% neutral buffered formalin and pupil-optic nerve cross sections were prepared. Serial pupil-optic nerve cross sections were cut in the horizontal plane (medial to lateral) where the superior oblique muscle could be identified. 10 µm paraffin-embedded sections were stained with hematoxylin and eosin, anti-p-tau (AT8, Thermo Fisher; 1:1000), anti-TDP43 (Proteintech, 10782-2-AP; 1:6000), and anti-p-TDP43 (phospho Ser409 pAb, Cosmo Bio; 1:1000) antibodies. For quantification of the thickness of the retinal ganglion cell layer, inner nuclear layer, and outer nuclear layer, we adapted the “retinal spider-gram” morphometric method used to quantify retinal degeneration in animal models [5, 10, 19, 20, 31]. The number of nuclei spanning the thickness of each lamina was counted manually at 1 mm and 2 mm distances from the optic nerve toward the ora serrata in both medial and lateral directions. Then, the means of retinal lamina nuclei at each distance in both directions were determined for each retina from the CTE and control cases. Student’s t-test was performed to determine if there were statistically significant differences in the means of retinal lamina thickness between the CTE and control eyes.

Brain tissue sections from subjects with Alzheimer’s disease, FTLD-TDP43 or normal brain autopsies were used as positive and negative immunohistochemistry controls for anti-p-tau, anti-TDP43, and anti-phosphorylated TDP43 antibodies as appropriate. In brief, for manual IHC using the anti-TDP43 antibody, slides were deparaffinized in xylene and rehydrated in graded ethanols. Slides then were placed in a 30% H_2_O_2_ in methanol solution for 30 min, washed in 0.1 M *tris*(hydroxymethyl)aminomethane (TRIS) at pH 7.6, blocked with 2% fetal bovine serum (FBS) in 0.1 M TRIS, and then slides were incubated in primary antibody at 4 °C overnight. The second day, after washing with 1% TRIS solution and blocking with 2% FBS, slides were incubated in biotinylated horse anti-rabbit IgG secondary antibody at 1:1000 concentration (Vector laboratories, Burlingame California) for one hour at room temperature, then for an additional one hour in avidin/biotin-based peroxidase (Vector laboratories, Burlingame California). The chromogen used was Discovery purple (Ventana Indianapolis, IN) and slides were subsequently dehydrated, cleared in xylene, and cover slipped. Slides were stained for AT8 and p-TDP43 on a Ventana Discovery Ultra (Ventana Medical Systems, Tucson, AZ, USA). For p-TDP43 the optimal antigen retrieval was using CC1 (Tris-EDTA; pH 8.6) at 95 °C for 40 min whereas for AT8 no antigen retrieval was needed. Endogenous peroxidase was quenched by treatment with dilute H_2_O_2_ for 12 min at 37 °C. The p-TDP43 (1:1000) or AT8 (1:100) primary antibodies were incubated on the sections for 32 min at 37 °C followed by treatment with goat anti-rabbit (p-TDP43) or goat anti-mouse (AT8) HRP-polymer secondary (OmniMap; Ventana Medical systems) at 37 °C for 12 min. Antibody presence was visualized using a purple detection kit (Ventana Medical systems) as a chromogen for 20 min followed by hematoxylin counterstain. Slides were rinsed in Dawn detergent (to remove the liquid cover slip oil) in water, dehydrated through alcohol and xylene and cover slipped.

## Results

### Demographics and background information

Autopsy enucleation specimens were obtained from 8 individuals with CTE as well as 8 controls (see “Materials and methods” section). The subjects with CTE differed from the controls in that all 8 CTE subjects were male (2 controls were female), and the mean age of the CTE subjects was 77.5 compared to 69.6 for the controls. The neuropathologic features of the CTE cases and controls are listed in Tables 1 and 2, respectively. Two control subjects showed mild to moderate Alzheimer’s disease neuropathologic changes (Table 2) [21]. There was limited ophthalmic history available including no information about ante-mortem visual symptoms for any of the subjects. However, CTE and control eyes showed no histopathologic features of geographic atrophy (e.g., loss of RPE and retinal degeneration), retinal neovascularization, or glaucoma (e.g., optic disc cupping, retinal ganglion cell loss).

### CTE retinas show no overt retinal degeneration

**Fig. 1:**
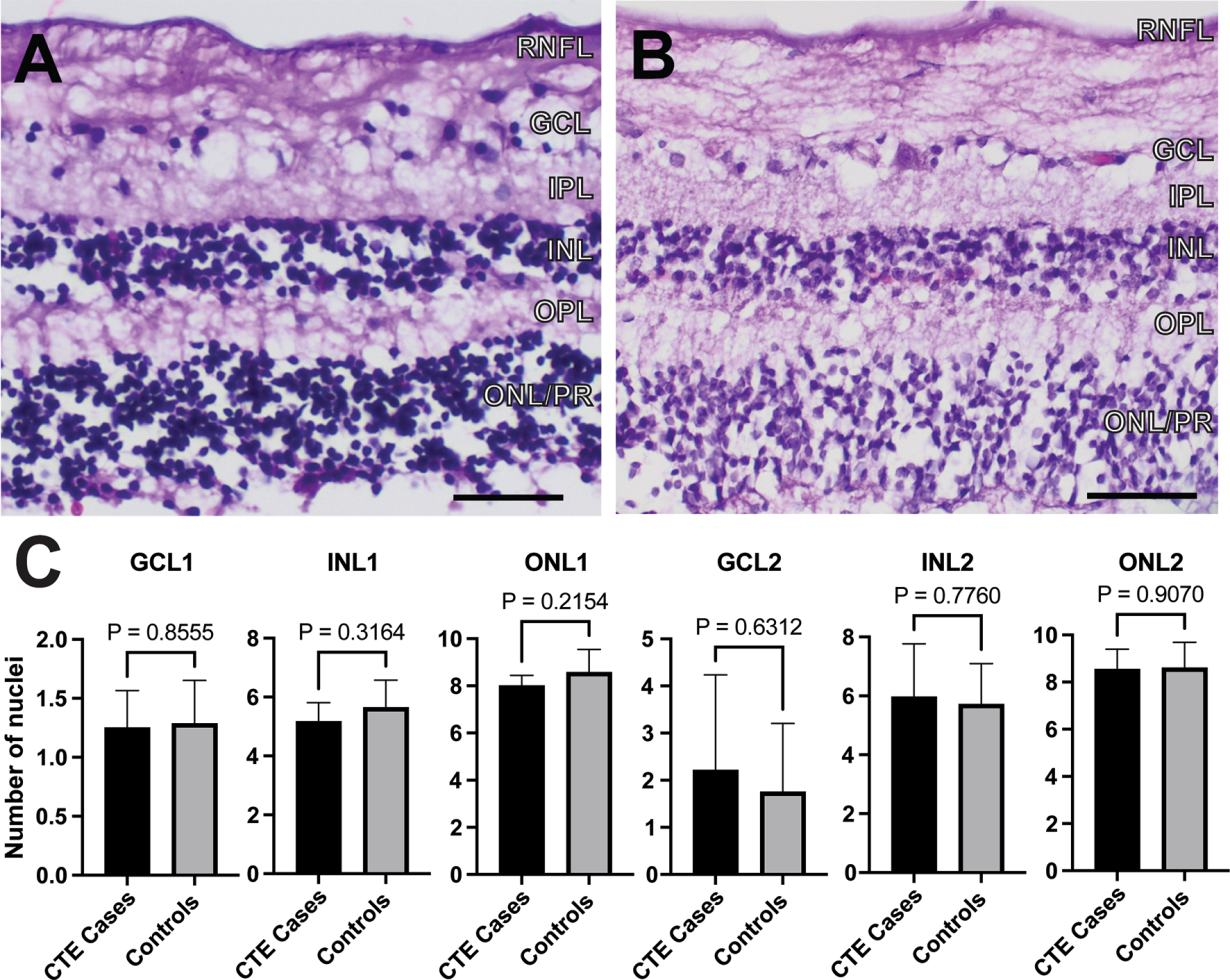
H&E. **A**, **B** Retinal thickness was comparable between control eyes (**A**) and CTE eyes (**B**); scale bar = 40 μm. **C** Quantification of retinal thickness in number of nuclei by layer, 1 mm (GCL1, INL1, ONL1) or 2 mm (GCL2, INL2, ONL2) from the optic nerve toward both equators per retina, in CTE (n = 6–7) and control eyes (n = 7). Error bars show mean ± standard errors. Student’s t-test comparisons of means between CTE and controls are shown, and *p* < 0.05 was considered statistically significant. RNFL, retinal nerve fiber layer, GCL, ganglion cell layer, IPL, inner plexiform layer, INL, inner nuclear layer, OPL, outer plexiform layer, ONL, outer nuclear layer

Pupil-optic nerve cross-sections of CTE eyes and control eyes were stained with H&E and evaluated for ocular pathology. All CTE eyes demonstrated intact retinal architecture and cellularity with no overt cell loss in cellular retinal layers (retinal pigment epithelium, outer nuclear layer, inner nuclear layer, and retinal ganglion cell layer) and no overt atrophy of non-cellular retinal layers (outer plexiform layer, inner plexiform layer, and retinal nerve fiber layer). Quantification of the thickness of individual retinal nuclear layers also revealed no statistically significant differences between CTE and control retinas (Fig. 1A–C).

### Many CTE retinas display abnormal phospho-TDP-43 aggregates in a subset of inner nuclear layer cells

**Fig. 2:**
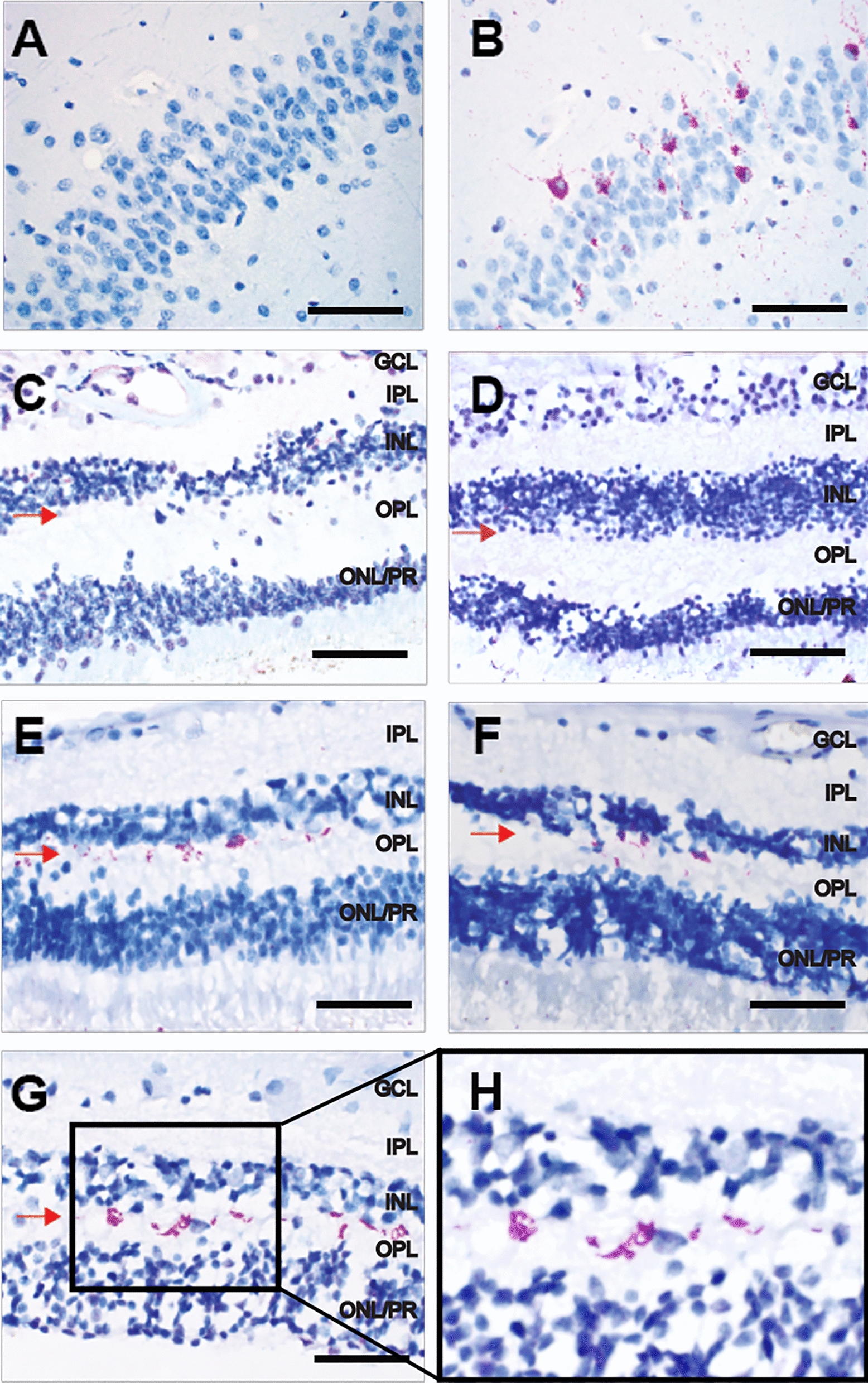
p-TDP43 immunohistochemistry. **A** Negative control—section of hippocampal dentate from a normal autopsy brain shows no p-TDP43 staining; scale bar = 80 μm. **B** Positive control—section of hippocampal dentate from patient with FTLD-TDP shows neuronal staining. **C**, **D** Representative control retinas show no p-TDP43 staining; scale bar = 60 μm. **E**–**H** Sections of retina from representative CTE cases show staining for p-TDP43 in outermost subset of cells (red arrow) in the inner nuclear layer. **H** Shows enlarged blow-up of area in square in (**G**); scale bar = 40 μm. All retina pictures are oriented with ONL at bottom

**Fig. 3:**
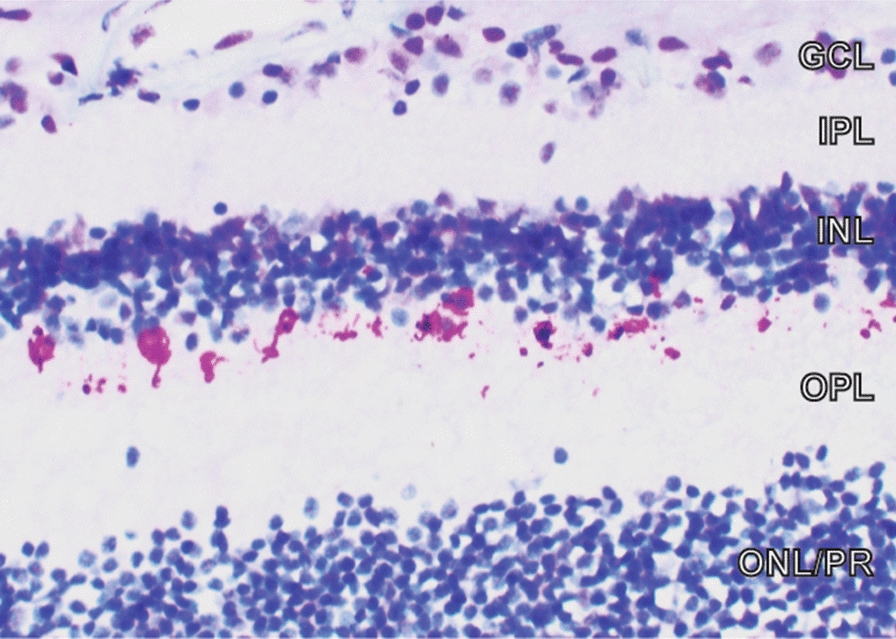
Positive p-TDP43 immunohistochemistry in control retina. p-TDP43 immunohistochemistry of the control retina (case #1) with p-TDP43 staining in outermost subset of cells in the INL. ONL is oriented at the bottom

Seven of 8 control retinas were negative for p-TDP43 staining (Fig. 2C–D). Control case #1’s retina displayed scattered punctate neuronal cytoplasmic p-TDP43 staining localized to the inner nuclear layer (INL) (Fig. 3). This control subject (control case #1) had no known history of any neurodegenerative condition and no significant neuropathological abnormalities. In contrast, 6/8 CTE retinas (all CTE cases except 4 and 7) displayed scattered punctate neuronal cytoplasmic p-TDP43 inclusions localized to the INL (Fig. 2E–H). The anatomic location and morphology of pTDP-43 positive cells in the INL most resembled horizontal cells, a subtype of INL interneuron whose cell bodies are located in the outermost aspect of the INL neighboring the outer plexiform layer (OPL), and whose neuritic processes extend in the same orientation (“horizontal”) as the INL retinal lamina. The density of neuronal cytoplasmic inclusions was similar between the 6 CTE retinas and the single control retina.

**Fig. 4:**
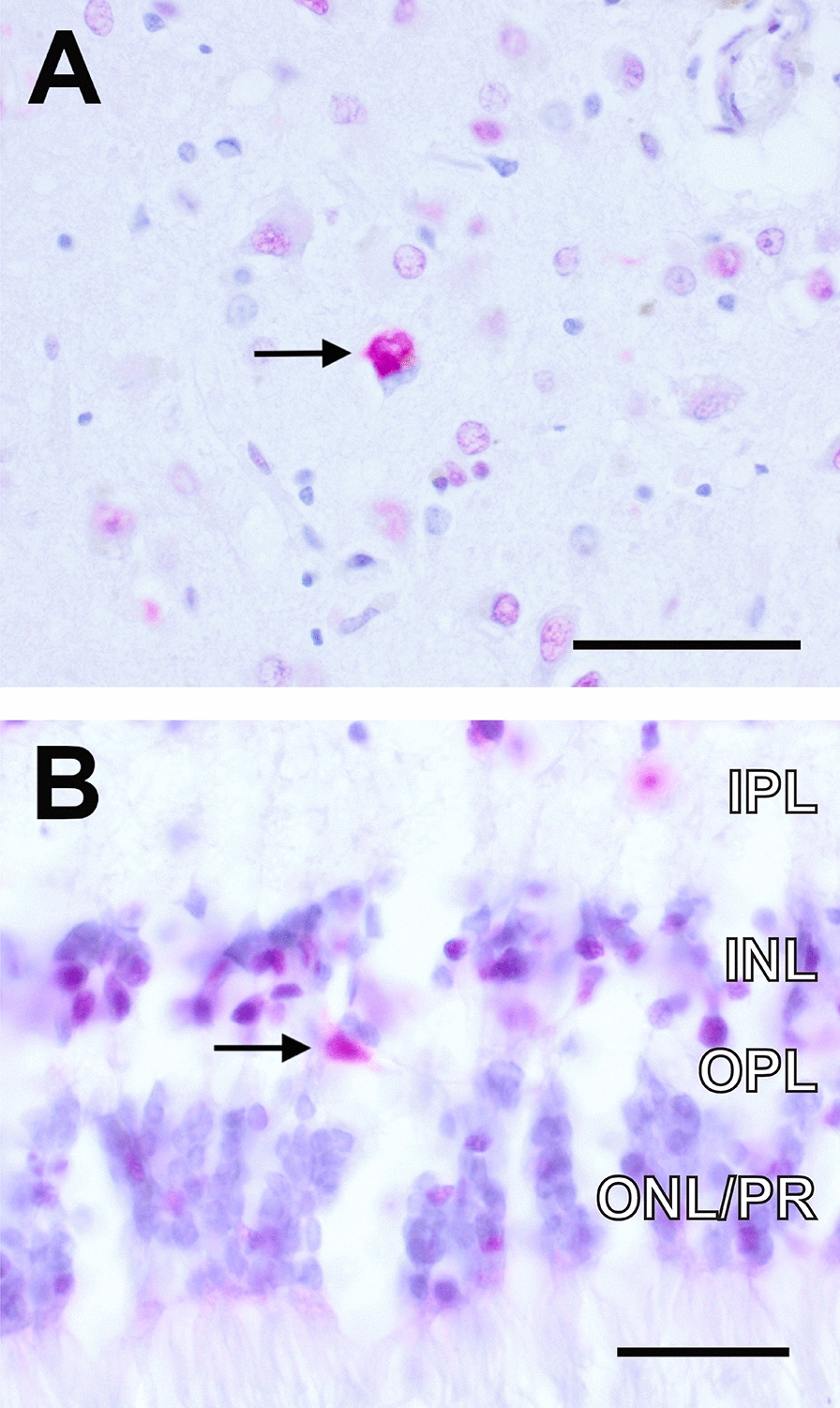
Total TDP43 immunohistochemistry. **A** Positive control—amygdala from a patient diagnosed with Alzheimer’s Disease, showing cytoplasmic positive, nuclear negative total TDP43 staining; scale bar = 50 μm. **B** CTE retina showing cytoplasmic positive, nuclear negative total TDP-43 staining of a cell (arrow) in the outermost aspect of inner nuclear layer; scale bar = 25 μm. Retina images are oriented with ONL at bottom

Total TDP43 staining also showed abnormal cytoplasmic inclusions in INL cells in the same 6/8 CTE retinas (Fig. 4A, B) and 1/8 control retinas, compatible with the abnormal p-TDP43 staining pattern seen in INL cells. Taken together, these results support that TDP43 pathology is common in CTE retinas and arises less frequently in non-CTE retinas (as in our control case #1). Interestingly, retinal TDP43 pathology in CTE is not ubiquitous in the retina but selectively targets cells of the INL, whose outward positions in the INL and neuritic morphologies are favored to represent horizontal cells.

### CTE retinas show no AT8 tau pathology

**Fig. 5:**
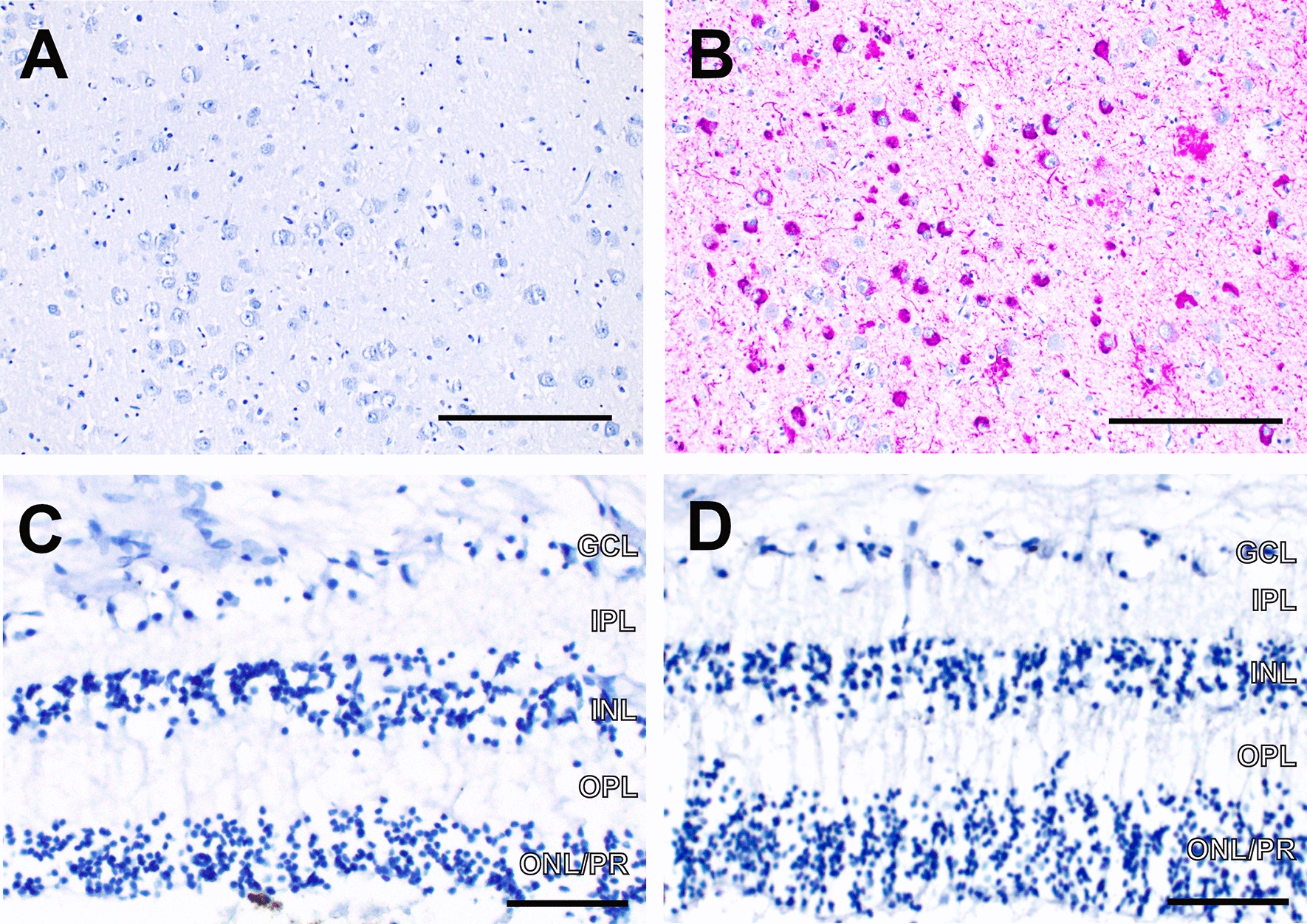
p-tau (AT8) immunohistochemistry. **A** Negative control—Frontal cortex from autopsy of a patient with no history of dementia; scale bar = 150 µm. **B** Positive control – Frontal cortex of Alzheimer’s disease patient; scale bar = 150 μm. **C** Control retina shows no p-tau (AT8) staining; scale bar = 60 μm. **D** Sections of retina from cases of CTE show no immunoreactivity for p-tau; scale bar = 60 μm

P-tau, an abnormal protein known to form aggregates in CTE and other neurodegenerative conditions [15], is a defining feature of CTE pathology in the brain [4, 24]. We observed no staining for p-tau using the monoclonal antibody AT8 in CTE or control retinas in any cellular or non-cellular retinal layers in serial pupil-optic nerve cross sections where we saw p-TDP43 retinal pathology (Fig. 5A–D).

## Discussion

CTE is a progressive neurodegenerative disorder, characterized by p-tau and p-TDP43 deposition in widespread regions of the central nervous system. This study is the first investigation of retinal pathology in CTE. In 6/8 cases of severe CTE, we found TDP43 pathology in the retina, consisting of scattered punctate cytoplasmic inclusions of p-TDP43 limited to INL cells. By contrast, similar p-TDP43 pathology was found in only 1/8 control retinas. In all positive cases, p-TDP43 retinal pathology was restricted to the INL retinal lamina in cells whose location and morphology were compatible with retinal horizontal cells but could also include other retinal cell types present in INL, including bipolar interneurons, Müller glia, and retinal vascular cells. Additional studies are important next steps to define cellular specificity, pathomechanism, and significance of p-TDP43 expression in the retina. By contrast, there was no AT8 p-tau pathology or overt evidence of neurodegeneration in the pupil-optic nerve cross sections from CTE retinas.

Given the role of horizontal cells in contrast enhancement and modulation of photoreceptor output, it is possible that p-TDP43 retinal pathology contributes to visual symptoms in individuals with CTE. Indeed, a previous case series documented a history of visuospatial symptoms in CTE patients including 7/13 patients with stage IV CTE and 18/45 patients with any stage of CTE [28]. However, the exact nature of visual symptoms and components of vision affected in CTE are not well-characterized. Horizontal interneurons are a regulatory cell type that provide feedback to photoreceptors [3, 34]. If p-TDP43 accumulation within horizontal interneurons causes cellular dysfunction, as it does in other neurons [8], this may affect modulation of visual stimuli leading to symptoms such as photosensitivity [29]. CTE pathology within other areas of the optic pathway could similarly lead to visuospatial anomalies. For instance, Armstrong et al. demonstrated that there is consistent involvement of the superior colliculi in CTE, as evidenced by p-tau immunoreactivity in 8 cases [2, 28]. However, except in the most severe cases of CTE, the primary visual cortex is typically spared [28].

One of our control retinas also showed focal accumulation of p-TDP43 with the same pattern of punctate cytoplasmic staining in putative INL horizontal cells seen in the CTE cases. Evaluation of this 81-year-old patient’s brain at autopsy showed no significant abnormalities. This patient had no known history of repetitive head trauma, consistent with the lack of brain pathology on autopsy, although we cannot exclude the possibility that this patient experienced some type of head or ocular injury which would lead to the presence of p-TDP43 in the retina. The presence and distribution of TDP43 or p-TDP43 in normal or diseased human retina has not been thoroughly investigated. Interestingly, a recent abstract described TDP43 inclusions in the retinas of 5/6 patients with FTLD (Dijkstra et al., 2021 Alzheimer’s & Dementia conference), which together with our current study suggests that TDP43 retinal pathology may be encountered in more disease settings.

Intriguingly, protein accumulations within CTE retinas were limited to p-TDP43, with no accumulations of p-tau identified, despite consistent p-TDP43 expression in INL. This suggests that although p-tau pathology is a defining feature of cerebral CTE, it is not a feature of retinal CTE. This is surprising, as the pathognomonic neuropathology findings of CTE include abnormal neuronal p-tau around small blood vessels at the base of cortical sulci [4, 24]. However, widespread TDP43 accumulation is also seen throughout the brain in late-stage CTE [26]. Why p-TDP43, but not p-tau, accumulates within the retina in stage IV CTE is unclear. One potential explanation is a difference in temporal regulation of CTE-associated markers, including p-tau, between the brain and the retina. In addition, two of the CTE retinas (ages 62 and 82, CTE cases 4 and 7) did not have p-TDP43, despite these subjects having positive TDP43 brain pathology. Finally, why there is no significant retinal degeneration (thinning of retinal cell layers) compared to the widespread neurodegeneration found in stage IV CTE brain is also unclear. Important caveats are that we only examined retinal pathology and did not examine optic nerves, because optic nerves were not removed during the enucleation process of CTE cases. Further, we examined pupil-optic nerve cross-sections collected at the horizontal (medial–lateral) axis. Also, we cannot exclude that retinas may express other phosphorylated tau species not recognized by the AT8 antibody used in our study.

In summary, this study is the first description of p-TDP43 pathology in CTE retinas and lays a foundation for further research into retinal dysfunction in visual deficits encountered in CTE patients, as well as p-TDP43 retinal pathology in other neurodegenerative conditions. However, there are several limitations of this study. First, there was a small cohort size for both the CTE and control groups, and further studies on larger cohorts will be needed to confirm our findings. Due to the retrospective nature of this study, we did not have complete clinical information regarding the patients’ visual status, limiting clinical-pathological correlation. In addition, the CTE patients included in this study all had stage IV disease, so we are not able to determine whether p-TDP43 pathology is a feature of severe CTE or of any stage of CTE. Finally, additional studies will be required to better characterize the cell types involved in CTE p-TDP43 retinal pathology. In particular, retinal cell type-specific molecular profiling will complement morphology and anatomy to determine if p-TDP43 expression is restricted to all horizontal cells; targets subsets of horizontal cells (e.g., H1, H2, H3, etc. [6]); or affects additional cell types in the INL (e.g., bipolar cells, Müller glia, retinal vascular cells) or other retinal laminae.

## Conclusions

In conclusion, p-TDP43 accumulation, but not p-tau, appears to be a common finding in late-stage (Stage IV) CTE retina. This retinal pathology may contribute to visual symptoms reported by CTE patients. Additional studies evaluating TDP43 within the retinas of CTE patients with lower stage disease (Stages I–III) are needed to elucidate the timing of this finding in relation to brain pathology. Furthermore, detailed visual examinations in patients with suspected CTE may be beneficial to better characterize visuospatial abnormalities and correlate these symptoms with both retinal and brain pathology.

## Data Availability

Data sharing is not applicable to this article as no datasets were generated or analyzed during the current study.

## Abbreviations

CTE: Chronic traumatic encephalopathy
H&E: Hematoxylin and eosin
GCL: Ganglion cell layer
INL: Inner nuclear layer
ONL: Outer nuclear layer
OPL: Outer plexiform layer
p-tau: Phosphorylated tau
p-TDP43: Phosphorylated transactive DNA-binding protein 43
TBI: Traumatic brain injury
TDP43: Transactive DNA-binding protein 43

## References

[1] Alosco ML, Cherry JD, Huber BR, Tripodis Y, Baucom Z, Kowall NW, Saltiel N, Goldstein LE, Katz DI, Dwyer B (2020). Characterizing tau deposition in chronic traumatic encephalopathy (CTE): utility of the McKee CTE staging scheme. Acta Neuropathol.

[2] Armstrong RA, McKee AC, Cairns NJ (2017). Pathology of the superior colliculus in chronic traumatic encephalopathy. Optom Vis Sci.

[3] Barnes S, Grove JCR, McHugh CF, Hirano AA, Brecha NC (2020). Horizontal cell feedback to cone photoreceptors in mammalian retina: novel insights from the GABA-pH hybrid model. Front Cell Neurosci.

[4] Bieniek KF, Cairns NJ, Crary JF, Dickson DW, Folkerth RD, Keene CD, Litvan I, Perl DP, Stein TD, Vonsattel JP (2021). The second NINDS/NIBIB consensus meeting to define neuropathological criteria for the diagnosis of chronic traumatic encephalopathy. J Neuropathol Exp Neurol.

[5] Chiang WC, Kroeger H, Sakami S, Messah C, Yasumura D, Matthes MT, Coppinger JA, Palczewski K, LaVail MM, Lin JH (2015). Robust endoplasmic reticulum-associated degradation of rhodopsin precedes retinal degeneration. Mol Neurobiol.

[6] Cowan CS, Renner M, De Gennaro M, Gross-Scherf B, Goldblum D, Hou Y, Munz M, Rodrigues TM, Krol J, Szikra T (2020). Cell types of the human retina and its organoids at single-cell resolution. Cell.

[7] Danielsen T, Hauch C, Kelly L, White CL (2021). Chronic traumatic encephalopathy (CTE)-type neuropathology in a young victim of domestic abuse. J Neuropathol Exp Neurol.

[8] de Boer EMJ, Orie VK, Williams T, Baker MR, De Oliveira HM, Polvikoski T, Silsby M, Menon P, van den Bos M, Halliday GM (2020). TDP-43 proteinopathies: a new wave of neurodegenerative diseases. J Neurol Neurosurg Psychiatry.

[9] den Haan J, Morrema THJ, Verbraak FD, de Boer JF, Scheltens P, Rozemuller AJ, Bergen AAB, Bouwman FH, Hoozemans JJ (2018). Amyloid-beta and phosphorylated tau in post-mortem Alzheimer's disease retinas. Acta Neuropathol Commun.

[10] Faktorovich EG, Steinberg RH, Yasumura D, Matthes MT, LaVail MM (1992). Basic fibroblast growth factor and local injury protect photoreceptors from light damage in the rat. J Neurosci.

[11] Goldstein LE, Fisher AM, Tagge CA, Zhang XL, Velisek L, Sullivan JA, Upreti C, Kracht JM, Ericsson M, Wojnarowicz MW (2012). Chronic traumatic encephalopathy in blast-exposed military veterans and a blast neurotrauma mouse model. Sci Transl Med.

[12] Goodwill VS, Dryden I, Choi J, De Lillo C, Soldau K, Llibre-Guerra J, Sanchez H, Sigurdson CJ, Lin JH (2022). Minimal change prion retinopathy: morphometric comparison of retinal and brain prion deposits in Creutzfeldt-Jakob disease. Exp Eye Res.

[13] Head MW, Northcott V, Rennison K, Ritchie D, McCardle L, Bunn TJ, McLennan NF, Ironside JW, Tullo AB, Bonshek RE (2003). Prion protein accumulation in eyes of patients with sporadic and variant Creutzfeldt-Jakob disease. Invest Ophthalmol Vis Sci.

[14] Ho CY, Troncoso JC, Knox D, Stark W, Eberhart CG (2014). Beta-amyloid, phospho-tau and alpha-synuclein deposits similar to those in the brain are not identified in the eyes of Alzheimer's and Parkinson's disease patients. Brain Pathol.

[15] Iqbal K, Liu F, Gong CX (2016). Tau and neurodegenerative disease: the story so far. Nat Rev Neurol.

[16] Kiernan PT, Montenigro PH, Solomon TM, McKee AC (2015). Chronic traumatic encephalopathy: a neurodegenerative consequence of repetitive traumatic brain injury. Semin Neurol.

[17] Koronyo-Hamaoui M, Koronyo Y, Ljubimov AV, Miller CA, Ko MK, Black KL, Schwartz M, Farkas DL (2011). Identification of amyloid plaques in retinas from Alzheimer's patients and noninvasive in vivo optical imaging of retinal plaques in a mouse model. Neuroimage.

[18] Lakis N, Corona RJ, Toshkezi G, Chin LS (2013). Chronic traumatic encephalopathy—neuropathology in athletes and war veterans. Neurol Res.

[19] LaVail MM, Gorrin GM, Repaci MA, Thomas LA, Ginsberg HM (1987). Genetic regulation of light damage to photoreceptors. Invest Ophthalmol Vis Sci.

[20] Lee EJ, Chan P, Chea L, Kim K, Kaufman RJ, Lin JH (2021). ATF6 is required for efficient rhodopsin clearance and retinal homeostasis in the P23H rho retinitis pigmentosa mouse model. Sci Rep.

[21] Lee J, Kim HJ (2022). Normal aging induces changes in the brain and neurodegeneration progress: review of the structural, biochemical, metabolic, cellular, and molecular changes. Front Aging Neurosci.

[22] Ling H, Morris HR, Neal JW, Lees AJ, Hardy J, Holton JL, Revesz T, Williams DD (2017). Mixed pathologies including chronic traumatic encephalopathy account for dementia in retired association football (soccer) players. Acta Neuropathol.

[23] Maroon JC, Winkelman R, Bost J, Amos A, Mathyssek C, Miele V (2015). Chronic traumatic encephalopathy in contact sports: a systematic review of all reported pathological cases. PLoS ONE.

[24] McKee AC, Cairns NJ, Dickson DW, Folkerth RD, Keene CD, Litvan I, Perl DP, Stein TD, Vonsattel JP, Stewart W (2016). The first NINDS/NIBIB consensus meeting to define neuropathological criteria for the diagnosis of chronic traumatic encephalopathy. Acta Neuropathol.

[25] McKee AC, Cantu RC, Nowinski CJ, Hedley-Whyte ET, Gavett BE, Budson AE, Santini VE, Lee HS, Kubilus CA, Stern RA (2009). Chronic traumatic encephalopathy in athletes: progressive tauopathy after repetitive head injury. J Neuropathol Exp Neurol.

[26] McKee AC, Gavett BE, Stern RA, Nowinski CJ, Cantu RC, Kowall NW, Perl DP, Hedley-Whyte ET, Price B, Sullivan C (2010). TDP-43 proteinopathy and motor neuron disease in chronic traumatic encephalopathy. J Neuropathol Exp Neurol.

[27] McKee AC, Robinson ME (2014). Military-related traumatic brain injury and neurodegeneration. Alzheimers Dement.

[28] McKee AC, Stern RA, Nowinski CJ, Stein TD, Alvarez VE, Daneshvar DH, Lee HS, Wojtowicz SM, Hall G, Baugh CM (2013). The spectrum of disease in chronic traumatic encephalopathy. Brain J Neurol.

[29] Merezhinskaya N, Mallia RK, Park D, Millian-Morell L, Barker FM (2021). Photophobia associated with traumatic brain injury: a systematic review and meta-analysis. Optom Vis Sci.

[30] Mez J, Daneshvar DH, Kiernan PT, Abdolmohammadi B, Alvarez VE, Huber BR, Alosco ML, Solomon TM, Nowinski CJ, McHale L (2017). Clinicopathological evaluation of chronic traumatic encephalopathy in players of American Football. JAMA.

[31] Michon JJ, Li ZL, Shioura N, Anderson RJ, Tso MO (1991). A comparative study of methods of photoreceptor morphometry. Invest Ophthalmol Vis Sci.

[32] Nicks R, Clement NF, Alvarez VE, Tripodis Y, Baucom ZH, Huber BR, Mez J, Alosco ML, Aytan N, Cherry JD (2023). Repetitive head impacts and chronic traumatic encephalopathy are associated with TDP-43 inclusions and hippocampal sclerosis. Acta Neuropathol.

[33] Samadani U, Ritlop R, Reyes M, Nehrbass E, Li M, Lamm E, Schneider J, Shimunov D, Sava M, Kolecki R (2015). Eye tracking detects disconjugate eye movements associated with structural traumatic brain injury and concussion. J Neurotrauma.

[34] Stroh S, Puller C, Swirski S, Holzel MB, van der Linde LIS, Segelken J, Schultz K, Block C, Monyer H, Willecke K (2018). Eliminating glutamatergic input onto horizontal cells changes the dynamic range and receptive field organization of mouse retinal ganglion cells. J Neurosci.

[35] Takao M, Kimura H, Kitamoto T, Mihara B (2018). PrP(res) deposition in the retina is a common finding of sporadic, familial and iatrogenic Creutzfeldt-Jakob diseases (CJD). Acta Neuropathol Commun.

[36] Ventura RE, Balcer LJ, Galetta SL (2014). The neuro-ophthalmology of head trauma. Lancet Neurol.

[37] Williams EA, McGuone D, Frosch MP, Hyman BT, Laver N, Stemmer-Rachamimov A (2017). Absence of Alzheimer disease neuropathologic changes in eyes of subjects with Alzheimer disease. J Neuropathol Exp Neurol.

